# Is Categorization in Visual Working Memory a Way to Reduce Mental Effort? A Pupillometry Study

**DOI:** 10.1111/cogs.13194

**Published:** 2022-09-07

**Authors:** Cherie Zhou, Monicque M. Lorist, Sebastiaan Mathôt

**Affiliations:** ^1^ Department of Experimental Psychology University of Groningen; ^2^ Faculty of Medical Sciences, Biomedical Sciences of Cells & Systems, Neurosciences, Neuroimaging Center University of Groningen

**Keywords:** Visual working memory, Categorization, Pupil dilation, Mental effort, Categorical representations, Mixture model

## Abstract

Recent studies on visual working memory (VWM) have shown that visual information can be stored in VWM as continuous (e.g., a specific shade of red) as well as categorical representations (e.g., the general category red). It has been widely assumed, yet never directly tested, that continuous representations require more VWM mental effort than categorical representations; given limited VWM capacity, this would mean that fewer continuous, as compared to categorical, representations can be maintained simultaneously. We tested this assumption by measuring pupil size, as a proxy for mental effort, in a delayed estimation task. Participants memorized one to four ambiguous (boundaries between adjacent color categories) or prototypical colors to encourage continuous or categorical representations, respectively; after a delay, a probe indicated the location of the to‐be‐reported color. We found that, for memory load 1, pupil size was larger while maintaining ambiguous as compared to prototypical colors, but without any difference in memory precision; this suggests that participants relied on an effortful continuous representation to maintain a single ambiguous color, thus resulting in pupil dilation while preserving precision. Strikingly, this effect gradually inverted, such that for memory load 4, pupil size was smaller while maintaining ambiguous and prototypical colors, but memory precision was now substantially reduced for ambiguous colors; this suggests that with increased memory load participants increasingly relied on categorical representations for ambiguous colors (which are by definition a poor fit to any category). Taken together, our results suggest that continuous representations are more effortful than categorical representations and that very few continuous representations (perhaps only one) can be maintained simultaneously.

## 1 Introduction

Visual information can be stored in visual working memory (VWM) as a detailed, continuously varying representation, such as a specific shade of green, or as a discrete, categorical representation, such as a prototypical green color. Traditionally, studies on VWM storage have focused on continuous representations. For example, one of the most commonly used analysis techniques in VWM research is the “mixture model” introduced by Zhang and Luck (2008). In this model, VWM performance is described as a mix of non‐random responses that are centered on the memorized color with some random error (i.e., a von Mises distribution) and random responses (guesses) that are represented by a uniform distribution. Although this model provides a good way to quantify some characteristics of VWM storage, in its original form it does not take category boundaries or prototypes into account and, therefore, implicitly assumes that all representations are continuous.

Recent studies have improved this by incorporating categorical representations into the mixture model (Bae & Luck, 2019; Hardman, Vergauwe, & Ricker, 2017; Panichello, DePasquale, Pillow, & Buschman, 2019; Pratte, Park, Rademaker, & Tong, 2017; Souza & Skora, 2017; Zhou et al., 2022). For example, Hardman et al. (2017) asked participants to memorize one or more colors and to report the memorized color after a delay period. To analyze their results, the authors used a “revised mixture model,” which included a parameter that reflects the proportion of VWM items that are stored in continuous or categorical representations. Crucially, they found that only one continuous representation can be stored in VWM at a time, whereas multiple categorical representations can be stored simultaneously. In other words, their results suggest that VWM capacity for continuous representations is far more limited than for categorical representations.

The distinction between continuous and categorical representations is related to the emerging view of VWM as relying on different brain areas that are involved in different aspects of VWM maintenance (Christophel, Klink, Spitzer, Roelfsema, & Haynes, 2017); specifically, continuous information would be represented predominantly in sensory areas, such as early visual cortex (Gayet et al., 2017; Hallenbeck, Sprague, Rahmati, Sreenivasan, & Curtis, 2021; Harrison & Tong, 2009; but see Ester, Sprague, & Serences, 2020) whereas categorical information would be represented predominantly in non‐sensory areas, such as parietal and prefrontal cortex, which are associated with learning and encoding categories at a more abstract level (Freedman, Riesenhuber, Poggio, & Miller, 2001; Lee, Kravitz, & Baker, 2013; but see Ester et al., 2015). This involvement of multiple brain areas with diverse functions means that VWM can flexibly adopt different representational formats, such as continuous or categorical, to store visual information depending on the needs of the situation.

Although previous studies have shown *that* VWM can rely on both categorical and continuous representations, it is still an open question *why* VWM uses both types of representations. After all, if VWM is able to store a detailed representation, then why would people ever sacrifice this level of precision by resorting to a lower resolution categorical representation? Recent studies have started to address the question of why VWM switches between continuous and categorical representations. For example, in a study by Bae and Luck (2019), participants maintained a single orientation in VWM, while performing a visual discrimination task during the delay period. Crucially, the authors found that when VWM maintenance was interrupted by the discrimination task, memory responses became less precise and more biased by the cardinal orientations (horizontal and vertical); that is, VWM representations became more categorical after a distracting task, possibly because the distraction consumed resources, thus reducing the resources available for VWM maintenance. This finding suggests that continuous representations, while more precise, are also more fragile than categorical representations.

In addition, as mentioned above, VWM representations are stored more categorically with increasing memory load (Hardman et al., 2017; Zhou et al., 2022). This suggests that VWM may have a lower capacity for continuous representations than for categorical representations; or, phrased differently, that it takes more resources to store a continuous representation than it does to store a categorical representation. (As we will get back to in the discussion, the first phrasing assumes a slot model of VWM, whereas the second phrasing assumes a resource model.) However, no study to date has directly tested this. Here we investigate this question by measuring pupil size while we encourage participants to maintain either continuous or categorical representations in VWM. Pupil dilation is commonly assumed to reflect mental effort during a range of cognitive tasks (Beatty & Lucero‐Wagoner, 2000; Laeng, Sirois, & Gredebäck, 2012; Mathôt, 2018; Unsworth & Robison, 2015), including working‐memory tasks (Kahneman & Beatty, 1966). For example, a recent study found that when participants held one or more color items in VWM for a subsequent change detection task, their pupils became larger with increasing memory load (Unsworth & Robison, 2015). In the present study, we predict that maintaining continuous representations in VWM will consume more mental effort than maintaining categorical representations, and therefore that pupils will be larger while maintaining continuous information as compared to categorical information.

To test this assumption, we included two experiments: In Experiment 1, we identified prototypes for seven common color categories, as well as boundaries between adjacent categories, using a category assignment procedure. In Experiment 2, we asked participants to memorize one or more colors and to reproduce one of the colors after a delay period. Crucially, the memory colors were either “ambiguous” (i.e., the category boundaries, such as a hue that sits perfectly in‐between red and orange) or prototypical (i.e., the category prototypes, such as a prototypical red hue), as identified in Experiment 1. We assumed that this manipulation would encourage the storage of continuous or categorical VWM representations for, respectively, ambiguous and prototypical colors. To reduce verbal encoding, we encouraged nonverbal representations by instructing participants to remember the exact colors and by providing visual feedback about their response accuracy after each trial. Our main prediction was that pupil size should increase while maintaining ambiguous as compared to prototypical colors. Moreover, we also predicted a traditional effect of memory load; that is, pupil size should increase with increasing memory load.

## 2 Experiment 1

The purpose of Experiment 1 was to identify the prototype for each of the seven basic color categories (Hardman et al., 2017) as well as the boundaries between categories. On each trial, we showed one of 360 hues (every 1° of the hue‐saturation‐value color circle, with full saturation and value) and asked participants to assign them to one of seven color labels. The resulting prototypes and boundary values will be used as prototypical and ambiguous colors, respectively, in Experiment 2.

## 3 Method

### 3.1 Participants

Thirty‐one first‐year psychology students (aged from 18 to 26 years old; 26 female, 5 male) from the University of Groningen participated in this experiment online in exchange for course credits. All participants had normal or corrected‐to‐normal acuity and color vision. The study was approved by the Ethics Committee of Psychology of the University of Groningen (PSY‐2021‐S‐0368).

### 3.2 Procedure, data processing, and results

**Fig. 1:**
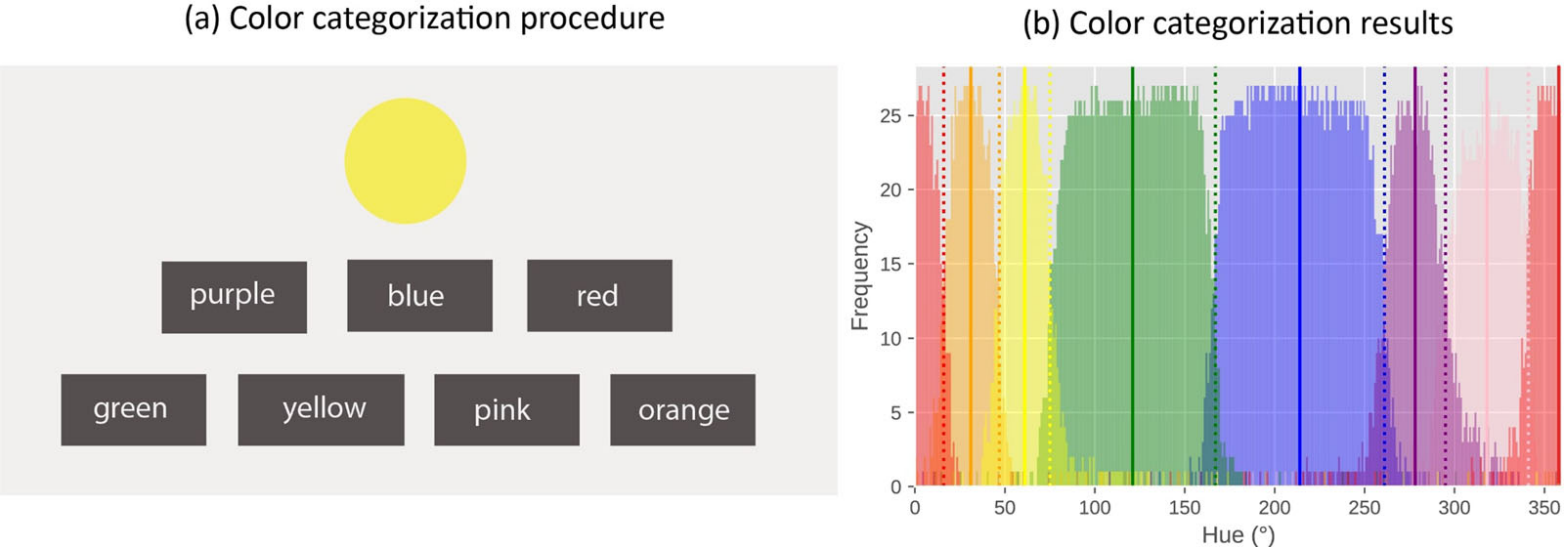
(a) An example of the color categorization procedure in Experiment 1. (b) Results of Experiment 1. Response frequency of each category is shown for each of the 360 hues. The center of each category (i.e., the prototypes) is marked by the vertical lines. The boundaries between categories are marked by the dotted lines. (Note: The category red is centered on 0° and is therefore split in this figure.

On each trial, a colored circle was shown in the center of the screen. The hues were chosen from an HSV (hue‐saturation‐value) color circle with full value (i.e., brightness) and saturation. Below the circle, seven color labels (red, pink, purple, blue, green, yellow, and orange) were shown horizontally (Fig. 1a). The order of the color labels was random on every trial. Participants were instructed to click or tap on the label that they thought this hue belonged to. The circle and the color labels remained on the screen until response. Three‐hundred‐sixty different hues (for each 1° of the color circle) were shown once during the experiment in random order.

Four participants whose responses strongly deviated from the average response (Fig. 1b) and who in some cases seemed to have responded randomly (see an example here: https://osf.io/kjpey/) were removed after visual inspection. The criterion for removal was subjective but was done before starting Experiment 2, to avoid “researchers degrees of freedom” while conducting the main analyses. Twenty‐seven participants remained for further analysis.

Next, we calculated the response frequency of each category for every hue value, starting from red. When the most frequently assigned category changed from one hue value to the next, this was chosen as the boundary between the two categories. In other words, boundary hues were maximally ambiguous in the sense that participants were equally likely to assign one of two color categories to them. The average of two nearby boundary values was chosen as the prototypes of the category (Fig. 1b; see details of response frequency of each category: https://osf.io/7sqne/).

## 4 Experiment 2

### 4.1 Method

#### 4.1.1 Participants

Thirty first‐year psychology students (between 18 and 23 years old; 22 female, 8 male) from the University of Groningen participated in exchange for course credits. All participants had normal or corrected‐to‐normal acuity and color vision. The study was approved by the Ethics Committee of Psychology of the University of Groningen (PSY‐2122‐S‐0014). Since we did not have an a priori estimate of an expected effect size, our sample size was not based on a power analysis. Rather, we relied on our lab default of testing 30 participants for within‐subject pupillometry studies that focused on VWM (e.g., Wilschut & Mathôt, 2022). This is comparable to the rule of thumb recently provided by Brysbaert and Stevens (2018) of having 1,600 observations per condition (we have slightly more observations when considering our main effects and slightly fewer when considering the full interaction).

### 4.2 Stimuli, design, and procedure

**Fig. 2:**
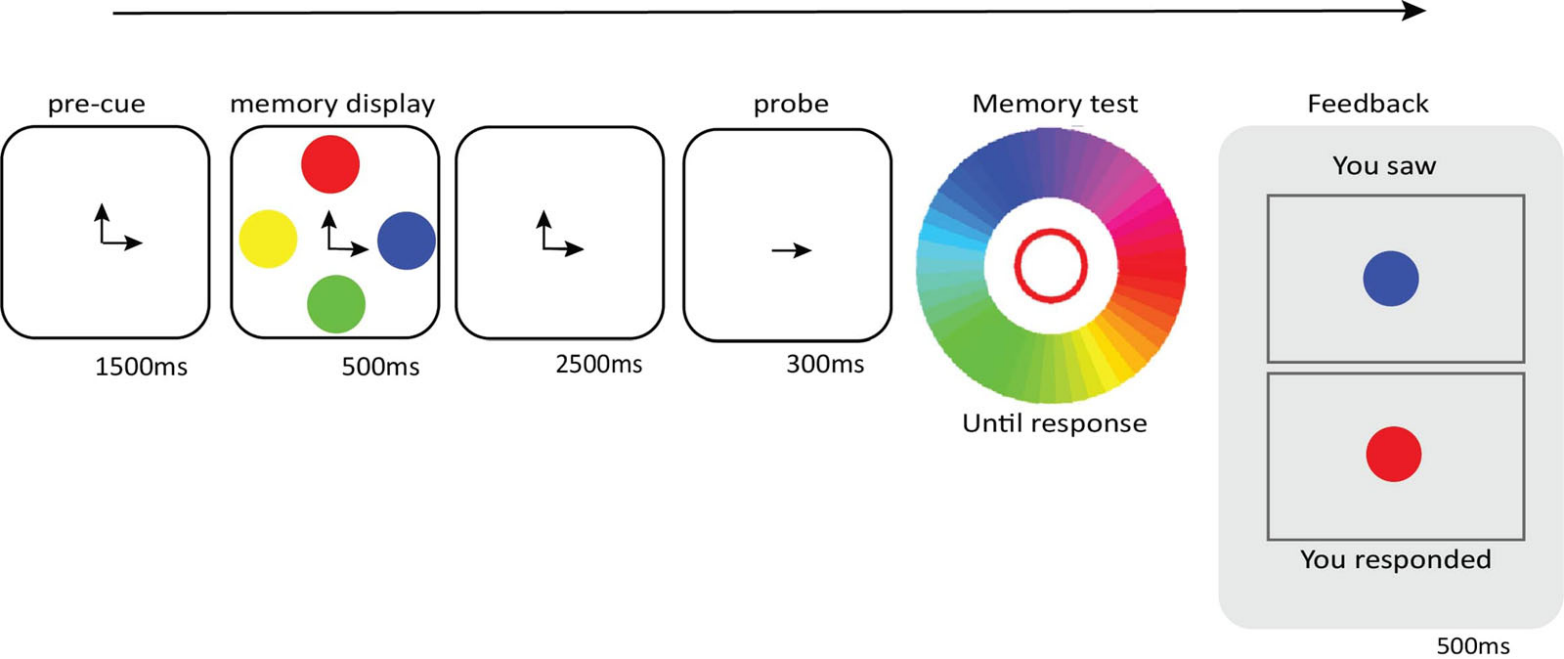
Sequence of events of a trial in Experiment 2 with a memory load of two and a prototypical color type.

Each trial started with a 1,500‐ms pre‐cue consisting of a number of arrows indicating the locations of the stimuli that need to be memorized (Fig. 2). The number of arrows varied depending on the memory load condition (one to four). These arrows remained in the subsequently presented memory display, which showed four colors. (There were always four colors in the memory display so that visual stimulation did not differ between different memory loads.) The memory colors were randomly drawn from a list of prototypical hue values from seven categories: red, pink, purple, blue, green, yellow, and orange; or from a list of seven ambiguous hue values that correspond to the boundaries between adjacent categories (e.g., red‐pink, blue‐green, etc.). The prototypes and the ambiguous colors were based on the values observed in Experiment 1. Next, following a 2,500‐ms retention interval, only one of the arrows from the previous displays remained on the screen for another 300 ms as a probe, indicating which color should be reproduced (i.e., the target color). Finally, participants reproduced the probed memory color on a color circle with no time limit. Visual feedback followed, comparing what they had selected with the color that had been presented at the cued location.

During the experiment, participants placed their chin on a head rest, at a distance of approximately 60 cm from the monitor. Pupil size was recorded monocularly (of the right eye) with an EyeLink 1000+ eye‐tracker (SR Research, Ontario, Canada), with a sampling rate of 1,000 Hz. Gaze positions were calibrated using a nine‐point calibration procedure. Before each trial, a drift correction was performed using a single‐point recalibration. The experiment was conducted in a dimly lit room with no distractors. Stimulus presentation and response collection were controlled with OpenSesame (version 3.3.5; Mathôt, Schreij, & Theeuwes, 2012) and PyGaze (Dalmaijer, Mathôt, & Van der Stigchel, 2014).

The memory load and color type conditions were mixed randomly within blocks without constraints. Participants completed 32 blocks of eight trials each (256 trials in total), preceded by one practice block of four trials.

### 4.3 Pre‐processing and exclusion criteria

We followed the pre‐processing guidelines as described in Mathôt and Vilotijević (2022). First, blinks in the pupil data were corrected using the “advanced” blink‐reconstruction mode in the Python library DataMatrix (0.13). Next, pupil data were baseline‐corrected by subtracting the mean pupil size during the first 100 ms of the memory display from the entire pupil waveform. (We also conducted a control analysis using the first 100 ms of the pre‐cue presentation as the baseline period, because, given that pupil responses have a latency of at least 200 ms, at this point neither the pre‐cue nor the memory display could have affected pupil size at all. The results were not dependent on the choice of baselines. All key results held under this analysis.) For each participant separately, we calculated the *z*‐scored baseline pupil size; trials in which the baseline deviated more than 2 standard deviations from the mean were excluded from the pupil size (but not the behavioral) analysis. In total, 348 trials were excluded based on this criterion. (The number of excluded trials per participant ranged from 8 to 17. See details here: https://osf.io/qvy6r/?pid=n9jgu.) Furthermore, we conducted a permutation test to test whether the mean accuracy of the participant was significantly above chance level. Specifically, we shuffled the response hue of all trials for each participant and decided the “shuffled absolute error” based on the distance between the shuffled response hue value and the target hue value on each trial. We then conducted an independent samples *t*‐test to test if the response error of each participant was significantly lower than the shuffled response error (with an alpha level of 0.05). No participants were excluded based on this criterion (i.e., all participants performed above chance).

## 5 Statistical analyses

### 5.1 Pupil size

For each 10 ms window separately, we conducted a linear mixed effects model linear mixed effects model (LMER) using the R package lmerTest (Bates, Kliegl, Vasishth, & Baayen, 2018) with pupil size as a dependent variable, memory load and color type as fixed effects, with random by‐participant intercepts and slopes for all fixed effects. We used an alpha level of 0.05 for at least 200 ms (i.e., 20 consecutive windows).

To properly control for multiple comparisons, we used fourfold, interleaved cross‐validation in combination with a linear‐mixed effects model (similar to the model described above) to identify the window during which the interaction effect was strongest. Finally, to qualify the interaction, we tested the color type effect for each memory load separately during this window by conducting four separate linear‐mixed effects analyses. (See details of the time series test: https://github.com/smathot/time_series_test)

### 5.2 Behavior: Precision and guess rate

First, we calculated the response error, which reflects the circular distance between the memory hue and the response hue. Next, we fitted a two‐parameter mixture model, using the biased‐memory toolkit (Zhou et al., 2022), to the distribution of response error, resulting in two parameters: precision [0, 10,000] and guess rate ([0, 1]). We conducted separate repeated measure analyses of variance (RM‐ANOVA) with memory load and color type as independent variables, and each of the two model parameters as dependent variables: precision and guess rate.

## 6 Results

### 6.1 Pupil size

**Fig. 3:**
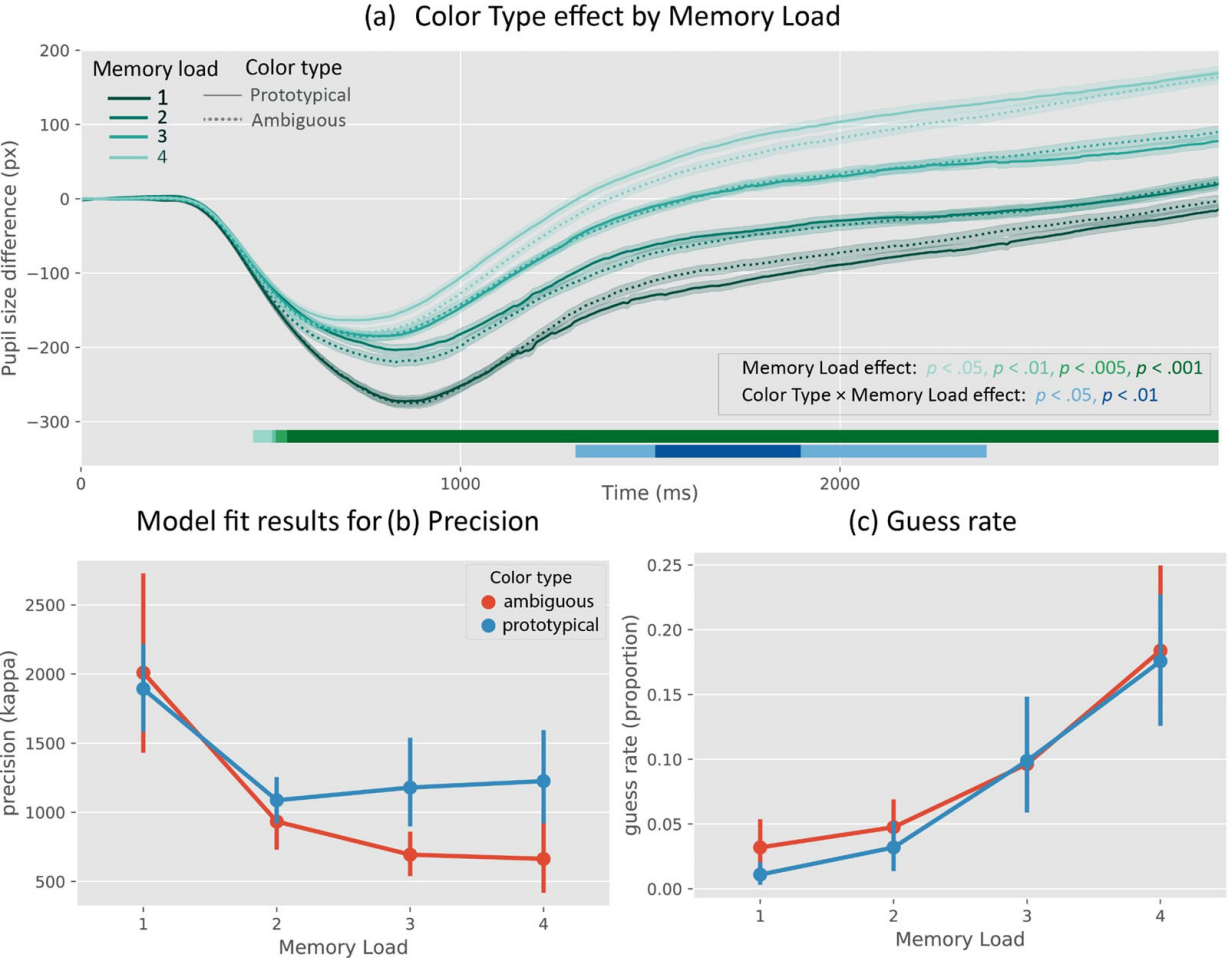
(a) Pupil size since the onset of the memory display as a function of color type and memory load. (b) Model fit results for precision and (c) guess rate as a function of color type and memory load.

As shown in Fig. 3a, there was an interaction between color type and memory load on pupil size (based on a criterion of *p* < .05 for at least a 200‐ms contiguous interval as described above). This effect started at 1,290 ms and remained until 2,140 ms after the start of the retention interval.

Our cross‐validation analysis similarly revealed an interaction, which was strongest at samples 1500 and 1650 (*z* = 2.52, *p* = .01). Follow‐up tests using mean pupil size during the 1,500–1,650 ms window showed that, at memory load 1, pupils were larger when participants memorized ambiguous colors, compared to prototypical colors (*z* = −2.42, *p* = .02); at memory load 2 and 3, there was no difference in pupil size between the ambiguous and prototypical colors (*z* = 1.14, *p* = .26; *z* = 0.16, *p* = .88); however, at memory load 4, pupils were smaller when participants memorized ambiguous colors, compared to prototypical colors (*z* = 2.22, *p* = .03).

In addition, there was a strong memory load effect on pupil size that arose 500 ms after memory stimuli onset and remained throughout the retention interval.

### 6.2 Behavior: Precision, guess rate, and response distributions

For precision, there was an effect of color type (*F*(1,29) = 5.21, *p* = .03), such that precision was higher for prototypical, as compared to ambiguous colors. Interestingly, we found an interaction effect between color type and memory load (*F*(3,87) = 3.17, *p* = .03; as shown in Fig. 3b); specifically, at memory load 1 and 2, there was no difference in precision between ambiguous and prototypical conditions (1: *t*(29) = 0.58, *p* = .57; 2: *t*(29) = −2.02*, p* = .05); whereas at memory load 3 and 4, responses in the ambiguous condition were significantly less precise than in the prototypical condition (3: *t*(29) = −2.83, *p* < .01; 4: *t*(29) = −3.14, *p* < .01). This suggests that when memory load was low, VWM relied on continuous representations for ambiguous colors; however, when memory load increased, VWM resorted to categorical representations even for ambiguous colors, which is reflected by the low precision for ambiguous colors. Moreover, there was a strong effect of memory load on precision (*F*(3,87) = 19.07, *p* < .01), such that precision decreased with increasing memory load.

For guess rate, we found no effect of color type (*F*(1,29) = 1.08, *p* = .31) nor an interaction between color type and memory load (*F*(3,87) = 0.58, *p* = .63). However, we did find a strong effect of memory load on guess rate (*F*(3,87) = 32.64, *p* < .01), such that guess rate increased with increasing memory load (Fig. 3 *c*).

**Fig. 4:**
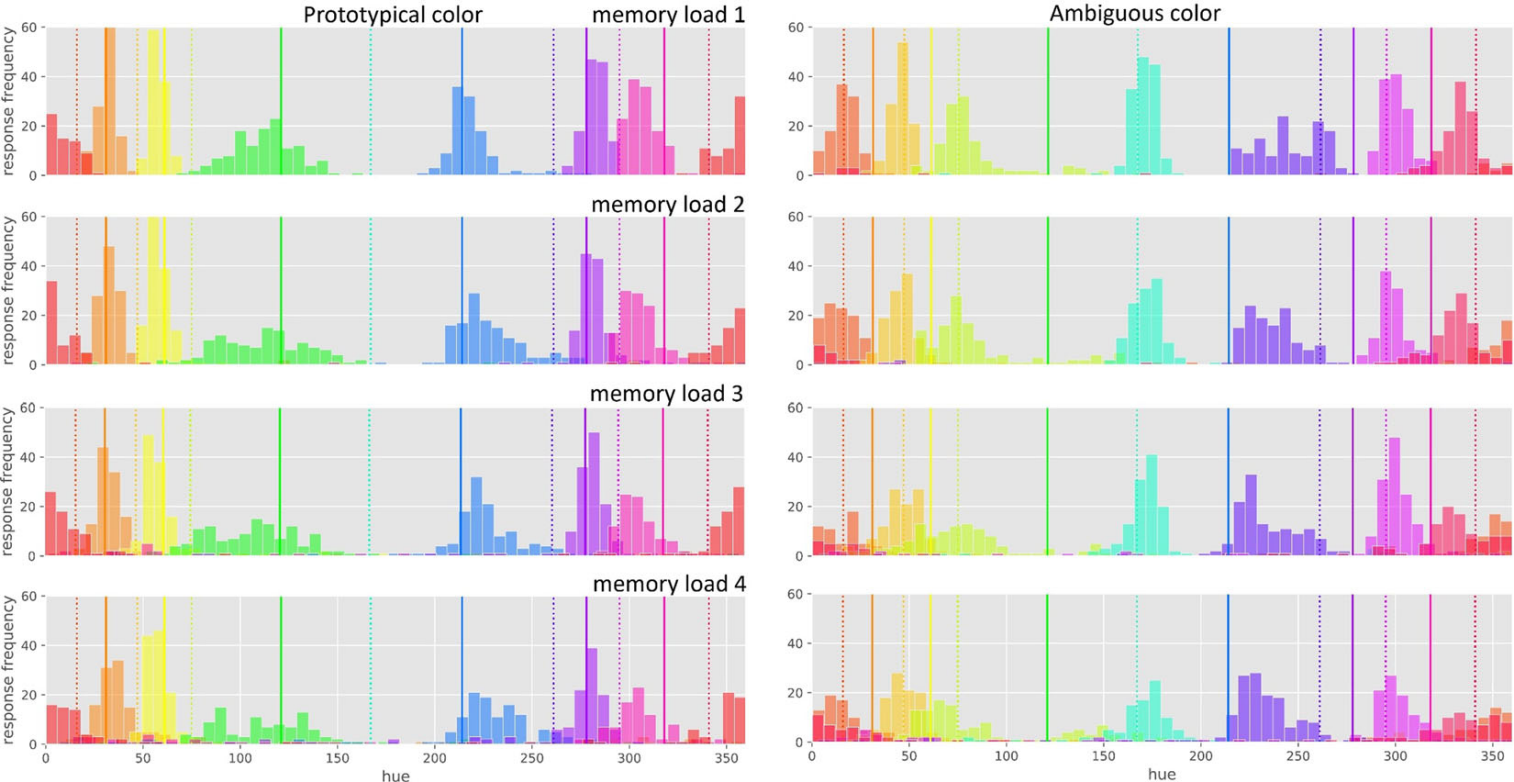
The response distributions for prototypical colors (on the left) and ambiguous colors (on the right) as a function of memory load (one to four). The *x*‐axis represents hues in degrees, according to the standard hue‐saturation‐value color circle, with 0° corresponding to red. Vertical solid lines indicate prototypical colors. Vertical dotted lines indicate ambiguous colors. The color of the response distributions indicates the memory color. For example, the blue response distribution around the solid blue vertical line in the top left indicates that participants were fairly precise in reproducing a prototypical blue color for memory load 1.

Fig. 4 shows response distributions for prototypical and ambiguous colors and for each memory load separately. These distributions, which are purely descriptive, illustrate in more detail what happens to color representations as memory load increases. For example, consider the prototypical blue color, which is indicated by the blue, vertical solid line in the left panel. For memory load 1, participants often reproduced this color accurately, as indicated by a peak in the distribution around the blue solid line; however, with increasing memory load, the blue response distribution becomes wider. This shows that prototypical colors were remembered less precisely as memory load increased. In contrast, for the ambiguous blue‐purple color, which is indicated by the blue‐purple, vertical dotted line in the right panel. For memory load 1, participants also reproduced this color accurately; however, with increasing memory load, the distribution shifts markedly towards the prototypical blue color, and in fact becomes *less* wide, as indicated by a peak in the blue‐purple response distribution around the blue, vertical solid line. A similar pattern is evident, though more subtle, for other ambiguous colors. This suggests that ambiguous colors were not only remembered less precisely with increasing memory load but that they were categorized (mainly as blue in the case of the blue‐purple ambiguous color), despite being a poor fit to any category.

## 7 Discussion

The present study investigated whether keeping continuous representations in VWM costs more mental effort than keeping categorical representations in VWM. To this end, we measured pupil size, as a proxy for mental effort, while participants maintained one or more prototypical or ambiguous colors in VWM; the prototypical colors corresponded to previously defined color‐category prototypes, whereas ambiguous colors corresponded to the boundaries between adjacent color categories. (Color‐category boundaries and prototypes had been determined in Experiment 1 using a separate group of participants.) The manipulation of prototypical versus ambiguous colors was designed to encourage categorical versus continuous representations, respectively.

We found that, for memory load 1, pupil size was larger in the ambiguous condition, as compared to the prototypical condition, presumably reflecting increased mental effort while maintaining a single continuous representation. For memory load 1, there was no difference in memory precision between the two color‐type conditions, suggesting that a continuous representation for a color that is difficult to categorize (e.g., a shade that sits on the boundary between blue and green) has a comparable fidelity to a categorical representation for a color that is easy to categorize (e.g., a prototypical shade of blue). In contrast, for memory load 2 and 3, there was no difference in pupil size between the ambiguous and prototypical conditions, and, for memory load 4, pupil size was actually smaller for ambiguous as compared to prototypical colors; however, precision for ambiguous colors was now substantially lower than that for prototypical colors. These results suggest that VWM increasingly relied on categorical representations with increasing memory load, even for ambiguous colors that were difficult to categorize, thus resulting in reduced precision for these colors. In addition, we found a traditional overall effect of memory load on pupil size, reflecting that even for categorical representations, mental effort increases with memory load.

These results can be interpreted slightly differently following two opposing models of VWM: the slot model (Fukuda, Awh, & Vogel, 2010; Pratte et al., 2017; Zhang & Luck, 2008) and the resource model (Bays & Husain, 2008; Bays, Catalao, & Husain, 2009). According to the slot model, the capacity of VWM is limited to a fixed number of slots, with fewer slots for continuous representations, as compared to categorical representations (e.g., Hardman et al., 2017); as a result, when memory load reached the maximal number of slots for continuous representations (i.e., a single one in the case of the current study), VWM resorted to categorical representations. In contrast, the continuous resource model suggests that there is a single resource that is distributed across items; following this model, continuous representations would consume more resources than categorical representations. Although our results can be partially interpreted by both models, they seem to be more consistent with recent proposals (e.g., Pratte et al., 2017) that combine characteristics of both models; that is, there are a limited number of slots for both continuous and categorical representations, and each slot has a variable precision.

Importantly, our findings are reminiscent of previous findings suggesting that only a single continuous representation can be maintained at a time (Hardman et al., 2017) and that, at least in some cases (Frătescu, Van Moorselaar, & Mathôt, 2019; Zhou, Lorist, & Mathôt, 2020), only a single VWM representation can guide attention at a time (McElree, 2001; Oberauer, 2002; Olivers, Peters, Houtkamp, & Roelfsema, 2011; van Moorselaar, Theeuwes, & Olivers, 2014). In turn, these findings may be related to a recent proposal that VWM information can be maintained in two states: within or outside the focus of (internal) attention (Bae & Luck, 2019; Cowan, 2005; Oberauer, 2002). When there is only a single visual item to be maintained, this item can be maintained inside the focus of attention, which presumably corresponds to activity in visual cortex (Bae & Luck, 2019; Foster, Bsales, Jaffe, & Awh, 2017; Harrison & Tong, 2009; Pasternak & Greenlee, 2005). Therefore, this item is stored as a rich, detailed representation in VWM. In turn, this corresponds to what we have referred to as a “continuous” representation in this paper. The advantage of continuous representations is likely that they contain more sensory detail; however, maintaining continuous representations, as compared to categorical representations, comes at the expense of increased mental effort. Therefore, when more than one item is maintained in VWM, the additional items are maintained outside of the focus of attention, which presumably does not correspond to active processing in visual brain areas, but to activity in non‐visual areas (e.g., prefrontal cortex: Bae & Luck, 2019; Goldman‐Rakic, 1995; or perhaps even to a type of “activity‐silent” representation: Stokes, 2015; Wolff, Jochim, Akyürek, & Stokes, 2017). As a result, these additional items are stored as abstract, less‐detailed representations in VWM. In turn, this corresponds to what we have referred to as “categorical” representations. The advantage of such categorical representations is likely that they consume little mental effort; however, this comes at the expense of low precision, especially for items that are difficult to categorize (such as the ambiguous colors in the current study). Taken together, in recent years several theories of VWM have been proposed that are different from one another in some ways, yet all share the underlying notion that not all VWM representations are alike, and that the more detailed/ sensory/ continuous a VWM representation is (a) the more it is related to guidance of attention; (b) the more it is related to activity in sensory brain areas; and (c) as the present results suggest, the more mental effort it requires. An important avenue for future research and theorization will be to merge these loosely related notions into a coherent framework.

This distinction between continuous and categorical representations is also related to the categorical‐coordinate account as proposed by Kosslyn (1994) and Kosslyn, Maljkovic, Hamilton, Horwitz, and Thompson (1995). On this account, visual inputs are encoded as both continuous (or “coordinate”) and categorical representations at the same time, although the extent to which each is involved varies depending on the situation (Gilbert Aubrey, Terry, Paul, & Ivry, 2006; Suegami, Aminihajibashi, & Laeng, 2014); continuous representations are predominantly involved in guiding attention and action, whereas categorical representations are predominantly involved in object recognition (Kosslyn, 1994). In the current study, both ambiguous and prototypical colors were likely encoded as both types of representations to some extent. At memory load 1, continuous representations were most strongly involved so as to guide attention more precisely. However, at higher loads, it became more difficult to encode the colors continuously; therefore, categorical representations were predominantly involved so as to keep VWM information intact.

The current findings were driven by the assumption that pupil size is a proxy for mental effort; however, pupil size also indexes other cognitive processes, such as surprise (e.g., Alamia, VanRullen, Pasqualotto, Mouraux, & Zenon, 2019) and uncertainty (de Berker et al., 2016; Urai, Braun, & Donner, 2017). For ambiguous colors, which were difficult to categorize, the unfamiliarity, stress, or difficulty in categorization likely had an influence on pupil size, compared to prototypical colors. Therefore, future research is needed to extend the current results using more intermediate colors between the boundaries and the prototypes as ambiguous colors (instead of the colors which are exactly on the boundaries) and other measures of mental effort.

Overall, our results suggest that only very few (perhaps just one) continuous representations can be maintained in VWM at a time and that maintaining continuous representations costs more mental effort than maintaining categorical representations. Crucially, when multiple items need to be maintained, VWM resorts to categorical representations, which are less precise, especially for items that are not easily categorizable (such as our ambiguous colors), yet cost less mental effort.

## Open practices statement

All experimental data and materials can be found on the OSF (Open Science Framework): https://osf.io/n9jgu/. The Python implementation of the biased‐memory toolbox can be found on GitHub: https://github.com/smathot/biased_memory_toolbox/. The implementation of the time series test can be found on GitHub: https://github.com/smathot/time_series_test.

### Open Research Badges

This article has earned Open Data and Open Materials badges. Data and materials are available at https://osf.io/n9jgu/.
